# MicroRNA-339-5p inhibits colorectal tumorigenesis through regulation of the MDM2/p53 signaling

**DOI:** 10.18632/oncotarget.2379

**Published:** 2014-08-21

**Authors:** Cen Zhang, Juan Liu, Xiaolong Wang, Rui Wu, Meihua Lin, Saurabh V. Laddha, Qifeng Yang, Chang S. Chan, Zhaohui Feng

**Affiliations:** ^1^ Department of Radiation Oncology, Rutgers Cancer Institute of New Jersey, Rutgers, State University of New Jersey, New Brunswick, NJ, USA; ^2^ Department of Breast Surgery, Qilu Hospital, Shandong University, Ji'nan, China; ^3^ Center for Systems Biology, Rutgers Cancer Institute of New Jersey, Rutgers, State University of New Jersey, New Brunswick, NJ, USA

**Keywords:** microRNA-339-5p, colorectal cancer, p53, migration, invasion, tumorigenesis

## Abstract

Tumor suppressor p53 plays a central role in tumor suppression. To ensure its proper function, the levels and activity of p53 are under a tight regulation in cells. MicroRNAs are short non-coding RNAs that play an important role in regulation of gene expression. Recently, microRNA-339-5p has been reported to be frequently down-regulated in colorectal cancer, and furthermore, its down-regulation is associated with poor prognosis in cancer patients, which strongly suggests a tumor suppressive function of microRNA-339-5p in colorectal cancer. In this study, we found that microRNA-339-5p directly represses the expression of MDM2, a key negative regulator of p53, through binding to MDM2 3′-UTR in colorectal cancer cells. Through the down-regulation of MDM2, microRNA-339-5p increases p53 protein levels and functions, including p53 transcriptional activity and p53-mediated apoptosis and senescence in response to stress. Furthermore, microRNA-339-5p inhibits the migration and invasion of colorectal cancer cells and the growth of colorectal xenograft tumors in a largely p53-dependent manner. Our results highlighted an important role of microRNA-339-5p in suppression of colorectal tumorigenesis, and also revealed that regulating the p53 function is an important mechanism for microRNA-339-5p in tumor suppression.

## INTRODUCTION

The tumor suppressor p53 and its signaling pathway play a central role in suppression of tumorigenesis, including colorectal cancer [1-3]. In response to stress, p53 is activated and accumulated in cells, which in turn transcriptionally regulates many target genes to initiate various cellular responses to maintain genomic stability and prevent tumorigenesis, including apoptosis, cell cycle arrest and senescence [1-3]. p53 also plays a critical role in inhibition of migration, invasion and metastasis of cancer cells [1, 2, 4]. To ensure its proper function in tumor suppression, the levels and activities of p53 are under a tight and complex regulation by many different regulators and mechanisms in cells [2, 5, 6]. MDM2, an E3 ubiquitin ligase, is the most critical negative regulator for p53. MDM2 binds to p53 and degrades p53 through ubiquitination to keep the p53 at a low level under the non-stress conditions [7, 8]. Importantly, MDM2 itself is a transcription target of p53. Thus, p53 and MDM2 form an auto-regulatory negative feedback loop, which is tightly controlled to allow the appropriate response of p53 to stress in cells [5, 6]. MDM2 is frequently overexpressed in various types of tumors, including colorectal cancer, which attenuates p53 function and promotes tumorigenesis [9-11].

MicroRNAs (miRNAs) are a class of endogenously expressed, small (20–25 nucleotides) non-coding regulatory RNA molecules in cells. MiRNAs bind to the 3′-untranslated regions (3′-UTRs) of mRNAs in a sequence-specific manner, reducing mRNA stability and inhibiting translation, and thereby negatively regulate the expression of genes at the posttranscriptional level [12-14]. Recently, emerging evidence has shown that miRNAs are an important component of the p53 signaling pathway in addition to protein-coding genes [15-17]. p53 positively or negatively regulates the expression of certain miRNAs, which in turn mediates p53 function. At the same time, miRNAs also regulate p53 levels and functions through regulating p53 or regulators for p53 in cells. For instance, miR-125b and miR-504 have been recently identified as the direct negative regulators of p53 [15, 18, 19]. miR-192, miR-194, miR-25, miR-32 and miR-661 have been reported to directly repress MDM2 expression to activate p53 in cells [20-22]. These findings demonstrate that as a new component of the p53 signaling pathway, miRNAs play an important role in regulating and/or mediating p53 function in tumor suppression. The expression of many miRNAs is frequently altered in tumors [23, 24]. The altered expression of certain miRNAs could lead to the disruption of the fine balance between miRNAs and the p53 signaling pathway, and thus attenuates p53 function and contributes to tumorigenesis [15, 17].

Recently, microRNA-339-5p (miR-339-5p) was reported to be frequently down-regulated in different types of cancers, including colorectal cancers, which is associated with cancer metastasis and poor prognosis in cancer patients [25-27]. In this study, we found that miR-339-5p directly represses MDM2 expression, which in turn increases p53 protein levels and enhances p53 functions in regulating apoptosis and senescence in colorectal cancer cells in response to stress. Furthermore, miR-339-5p inhibits the migration and invasion of colorectal cancer cells as well as the growth of colorectal xenograft tumors in mice largely through the up-regulation of p53 functions. These results demonstrated that the up-regulation of p53 function is an important mechanism by which miR-339-5p inhibits colorectal tumorigenesis.

## RESULTS

### MiR-339-5p down-regulates MDM2 expression through its binding to the 3′-UTR of MDM2

Recently, miRNA-339-5p was reported to be frequently down-regulated in colorectal cancer, and furthermore, its decreased expression is associated with poor prognosis in colorectal cancer patients, which strongly suggests a potential role of miR-339-5p in suppression of colorectal cancer [25]. However, its underlying mechanism is not well-understood. To further understand the mechanism by which miR-339-5p exhibits tumor suppressive function in colorectal cancer, we performed a computational search for the potential targets for miR-339-5p. MDM2 was identified as a potential target for miR-339-5p, which contains multiple putative binding sites for miR-339-5p in its 3′-UTR.

**Figure 1:**
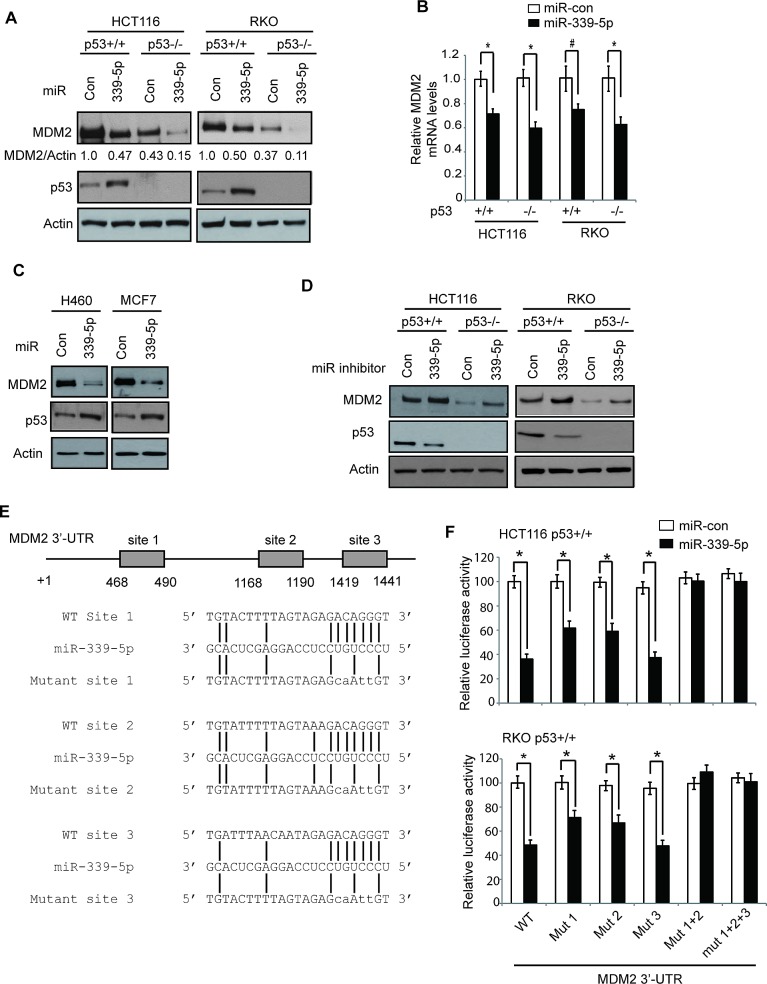
MiR-339-5p negatively regulates MDM2 levels in human colorectal cells through binding to human MDM2 3′-UTR (A) MiR-339-5p decreased MDM2 protein levels and increased p53 protein levels in human colorectal cancer HCT116 and RKO cells. HCT116 p53+/+, HCT116 p53−/−, RKO p53+/+ and RKO p53−/− cells were transfected with miR-339-5p mimic or scrambled miRNA control (miR-con), and the MDM2 and p53 protein levels were measured at 24 h after transfection by western-blot assays. (B) MiR-339-5p decreased MDM2 mRNA levels in HCT116 and RKO cells. The MDM2 mRNA levels were measured by Taqman real-time PCR in cells transfected with miR-339-5p mimic or miR-con, and normalized with actin. The levels of the MDM2 mRNA in control cells transfected with miR-con were designated as 1. Data are presented as mean ± SD (n=3). *#: p<0.05*, *: *p*<0.01, two-tailed Student *t*-tests. (C) MiR-339-5p decreased MDM2 protein levels and increased p53 protein levels in human lung H460 and breast MCF7 cells. (D) The miR-339-5p inhibitor increased MDM2 protein levels and reduced p53 protein levels in HCT116 and RKO cells. Cells were transfected with the miR-339-5p inhibitor or miR-con inhibitor, and the MDM2 and p53 protein levels were measured at 24 h after transfection by western-blot assays. (E) The sequences of miR-339-5p, its putative binding sites and their mutants in the 3′-UTR of human *MDM2*. The positions of putative binding sites are labeled. The drawing is not to scale. (F) miR-339-5p inhibited the luciferase activities of the luciferase reporter vectors containing the wild-type (WT) human *MDM2* 3′-UTR which includes the 3 putative binding sites. HCT116 p53+/+ and RKO p53+/+ cells were transfected with luciferase reporter vectors containing WT or different mutant human *MDM2* 3′-UTR together with miR-339-5p mimic or miR-con. Luciferase activities were measured at 24 h after transfection. Data are presented as mean ± SD (n = 3). *: *p*<0.01, two-tailed Student *t*-tests.

To examine whether miR-339-5p can regulate MDM2 protein levels, human colorectal cancer HCT116 p53+/+, HCT116 p53−/−, RKO p53+/+ and RKO p53−/− cells were transfected with miR-339-5p mimic or scrambled miRNA control (miR-con). These cell lines are two pairs of isogenic human colorectal cell lines with or without expression of wild-type (WT) p53, which have been widely used for p53 study [28-31]. MiR-339-5p mimic greatly reduced MDM2 protein levels in all these four colorectal cancer cell lines (Figure 1A). As a key negative regulator for p53, the down-regulation of MDM2 by miR-339-5p resulted in the induction of p53 protein in HCT116 p53+/+ and RKO p53+/+ cells. Furthermore, miR-339-5p mimic also reduced MDM2 mRNA levels in these cell lines, but to a less extent than its effect on MDM2 protein levels (Figure 1B). Interestingly, a stronger inhibitory effect of miR-339-5p on both MDM2 protein and mRNA was observed in HCT116 p53−/− and RKO p53−/− cells compared with HCT116 p53+/+ and RKO p53+/+ cells (Figure 1A,B). Since MDM2 is a direct transcriptional target of p53, the down-regulation of MDM2 by miR-339-5p could increase p53 protein levels and in turn transcriptionally induce MDM2, which could partially compromise the inhibitory effect of miR-339-5p on MDM2. In addition to the above-mentioned cell lines, the repression of MDM2 by miR-339-5p and the resultant induction of p53 protein levels were also observed in other cell lines, including human lung H460 cells and breast MCF7 cells which express WT p53 (Figure 1C), indicating that miR-339-5p represses MDM2 expression in different types of cells. To further confirm that miR-339-5p represses MDM2, the miR-339-5p inhibitor, single-stranded RNA oligonucleotides that completely match with mature miR-339-5p sequences, was used to treat HCT116 p53+/+, HCT116 p53−/−, RKO p53+/+ and RKO p53−/− cells. As shown in Figure 1D, the miR-339-5p inhibitor increased the MDM2 protein levels in all these cell lines, and reduced p53 protein levels in HCT116 p53+/+ and RKO p53+/+ cells. Taken together, these results demonstrated that miR-339-5p can negatively regulate MDM2 in cells.

Computational analysis predicted three putative binding sites for miR-339-5p located in the first 1.6 kb of 3′-UTR of the human *MDM2* mRNA (Figure 1E). To determine whether miR-339-5p binds to these three sites to down-regulate MDM2, a firefly luciferase reporter vector was constructed by inserting the first 1.6 kb of the 3′-UTR cDNA sequences of the human *MDM2* gene containing these three sites into the 3′ end of the luciferase gene. The vector was transfected into HCT116 p53+/+ or RKO p53+/+ cells together with either miR-339-5p mimic or miR-con. Compared with miR-con, miR-339-5p significantly decreased (by ~2-2.5-fold) the luciferase activities of the vectors containing the WT *MDM2* 3′-UTR sequences in both HCT116 p53+/+ and RKO p53+/+ cells (Figure 1F). We further constructed serial luciferase vectors containing the mutant MDM2 3′-UTR sequence by mutating different putative binding sites for miR-339-5p. As shown in Figure 1F, mutating either putative binding site 1 (Mut 1) or site 2 (Mut 2) partially rescued the luciferase activities reduced by miR-339-5p, whereas mutating the putative binding site 3 (Mut 3) alone failed to do so. Consistently, mutating the putative binding sites 1 and 2 together (Mut 1+2) almost completely abolished the inhibitory effect of miR-339-5p on the luciferase activities, whereas mutating these 3 putative binding sites together (Mut 1+2+3) did not further increase the luciferase activities. These results indicated that miR-339-5p targets *MDM2* through direct binding to the first two binding sites in *MDM2* 3′-UTR.

### MiR-339-5p increases p53 protein accumulation and its transcriptional activity in response to stress

**Figure 2:**
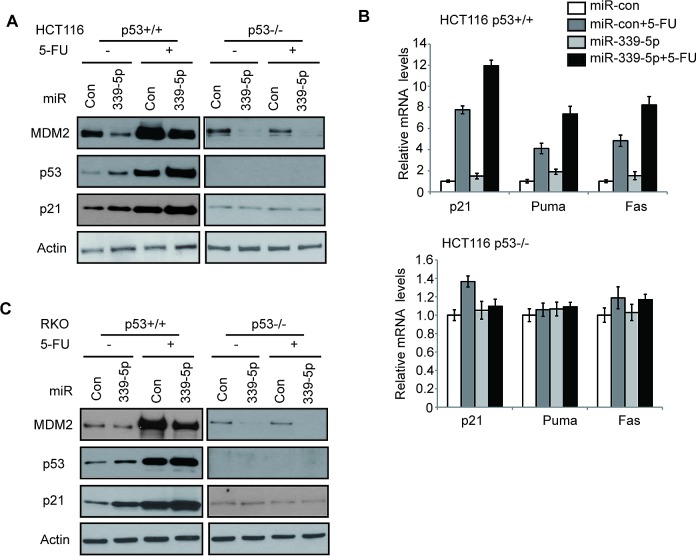
MiR-339-5p increases p53 protein accumulation and its transcriptional activity in response to stress by negatively regulating MDM2 in human colorectal cancer cells (A) MiR-339-5p decreased MDM2 protein levels and increased the p53 protein accumulation and transcriptional activity toward p21 in response to stress in HCT116 cells. (B) MiR-339-5p increased the p53 transcriptional activity toward p21, Puma and Fas in response to stress in HCT116 cells. In A and B: HCT116 p53+/+ and p53−/− cells were transfected with miR-339-5p mimic or miR-con. At 24 h after transfection, cells were treated with 5-FU (50 μM) for 8 h, and analyzed by western-blot (A) and Taqman real-time PCR (B), respectively. The mRNA levels of all genes were normalized to actin. The mRNA levels of genes in untreated cells transfected with miR-con were designated as 1. Data are presented as mean ± SD (n=3). (C) MiR-339-5p decreased MDM2 protein levels and increased p53, p21 protein levels in response to stress in RKO cells. RKO p53+/+ and RKO p53−/− cells were treated with 5-FU and analyzed as described in A.

p53 protein is maintained at a low level in cells under the non-stressed conditions mainly through proteasomal degradation of p53 protein by E3 ubiquitin ligases, particularly MDM2 [5, 6, 32]. In response to stress signals, p53 protein is accumulated in cells, which in turn leads to the transcriptional activation of p53 target genes to exert p53 functions in tumor suppression [1-3]. To investigate whether miR-339-5p reduces the p53 protein accumulation and p53 transcriptional activity in response to stress in colorectal cancer cells, HCT116 p53+/+ and HCT116 p53−/− cells transfected with miR-339-5p mimic were treated with chemotherapeutic agent 5-Fluorouracil (5-FU), which acted as stress signal to activate p53. 5-FU is the most widely-used chemotherapeutic agent for colorectal cancer [33, 34]. The miR-339-5p mimic induced p53 protein levels in HCT116 p53+/+ cells under both non-stressed and stressed conditions (5-FU treatment) (Figure 2A). Consistent with the increased p53 protein accumulation, the levels of p21 protein, a well-known p53 target, was also increased by miR-339-5p under both non-stressed and stressed conditions in a p53-dependent manner; p21 was clearly induced by miR-339-5p in p53+/+ but not p53−/− HCT116 cells (Figure 2A). This result demonstrated that the induction of p21 by miR-339-5p is due to the activation of p53 through MDM2 down-regulation. The increased p53 transcriptional activity by miR-339-5p was also confirmed by examining the mRNA levels of several p53 target genes, including p21 (involved in cell cycle and senescence), Puma and Fas (involved in apoptosis). MiR-339-5p induced the mRNA levels of these genes in HCT116 p53+/+ cells with or without 5-FU treatment as detected by real-time PCR assays (Figure 2B, upper panel). Furthermore, the mRNA levels of these genes were not affected by miR-339-5p in HCT116 p53−/− cells treated with or without 5-FU (Figure 2B, lower panel). In addition to HCT116 cells, miR-339-5p mimic reduced the MDM2 protein levels, which in turn increased the protein levels of p53 and p21, in RKO p53+/+ cells treated with or without 5-FU (Figure 2C). These results together showed that miR-339-5p enhances p53 protein accumulation and p53 transcriptional activity in response to stress in colorectal cancer cells.

### MiR-339-5p enhances p53 functions in mediating apoptosis and senescence in response to stress

**Figure 3:**
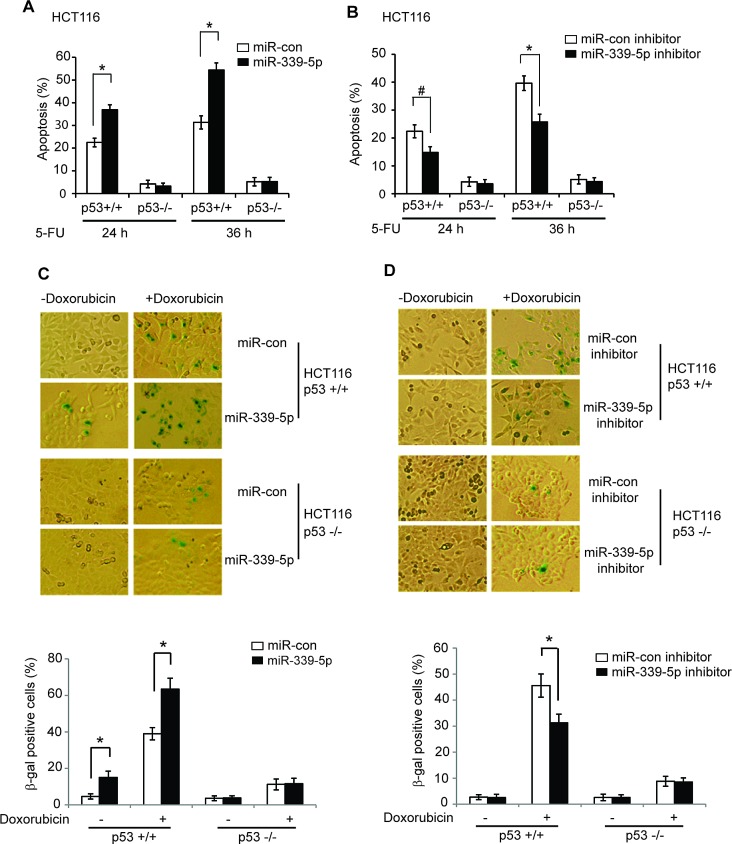
MiR-339-5p enhances p53-mediated apoptosis and senescence in response to stress (A) MiR-339-5p enhanced p53-mediated apoptosis in HCT116 cells treated with 5-FU. HCT116 p53+/+ and p53−/− cells transfected with miR-339-5p mimic or miR-con were treated with 5-FU (300 μM), and apoptosis were measured by Annexin V staining in a flow cytometer at 24 or 36 h after treatment. (B) The miR-339-5p inhibitor reduced p53-mediated apoptosis in HCT116 cells treated with 5-FU. HCT116 p53+/+ and p53−/− cells transfected with the miR-339-5p inhibitor or miR-con inhibitor were treated with 5-FU and analyzed as described in A. (C) MiR-339-5p enhanced p53-mediated senescence in HCT116 cells treated with Doxorubicin. HCT116 p53+/+ and p53−/− cells transfected with miR-339-5p mimic or miR-con were treated with 100 nM Doxorubicin for 3 days before cellular senescence was measured by SA-β-gal staining. (D) The miR-339-5p inhibitor reduced p53-mediated senescence in HCT116 cells treated with Doxorubicin. HCT116 p53+/+ and p53−/− cells transfected with the miR-339-5p inhibitor or miR-con inhibitor were treated with Doxorubicin and analyzed as described in C. In C and D: The upper panels are represented images of SA-β-gal staining. In A-D: Data are presented as mean ± SD (n = 3). *#: p<0.05*; *: *p*<0.01; two-tailed Student *t*-tests.

The p53-mediated apoptosis and senescence are the main mechanisms for p53 in tumor suppression [1, 2]. To investigate the impact of miR-339-5p upon p53-mediated apoptosis, HCT116 p53+/+ and p53−/− cells transfected with miR-339-5p mimic were treated with 5-FU, and cellular apoptosis was measured in a flow cytometer. It has been reported that 5-FU induces apoptosis in a largely p53-dependent manner in colorectal cancer cells, which is critical for colorectal cancer to respond to 5-FU-based chemotherapy [33, 34]. As shown in Figure 3A, the apoptosis induced by 5-FU was highly p53-dependent; much more cells underwent apoptosis in HCT116 p53+/+ cells than HCT116 p53−/− cells. Notably, the miR-339-5p mimic singificantly promoted 5-FU-induced apoptosis in HCT116 p53+/+ cells. In contrast, much less pronounced effect of miR-339-5p on 5-FU-induced apoptosis was observed in HCT116 p53−/− cells. Furthermore, the miR-339-5p inhibitor significantly decreased 5-FU-induced apoptosis in HCT116 p53+/+ cells but not in HCT116 p53−/− cells (Figure 3B). These results demonstrated that miR-339-5p enhances p53 function in mediating apoptosis in colorectal cells in response to stress.

It has been reported that chemotherapeutic agent Doxorubicin induces senescence in a largely p53-dependent manner in many different types of cell lines [35, 36]. To investigate whether miR-339-5p affects p53 function in inducing senescence, HCT116 p53+/+ and p53−/− cells transfected with the miR-339-5p mimic were treated with Doxorubicin, and senescent cells were detected by senescence associated β-galactosidase (SA-β-gal) staining. Doxorubicin induced senescence in a largely p53-dependent manner in HCT116 cells (Figure 3C, D), which is consistent with previous reports [35, 36]. Notably, the miR-339-5p mimic significantly increased Doxorubicin-induced senescence in HCT116 p53+/+ but not in HCT116 p53−/− cells (Figure 3C). Consistently, the miR-339-5p inhibitor significantly inhibited the Doxorubicin-induced senescence in HCT116 p53+/+ but not p53−/− cells (Figure 3D). These results clearly demonstrated that miR-339-5p promotes p53 function in mediating senescence in colorectal cells in response to stress.

### MiR-339-5p inhibits migration and invasion of colorectal tumor cells in a largely p53-dependent manner

**Figure 4:**
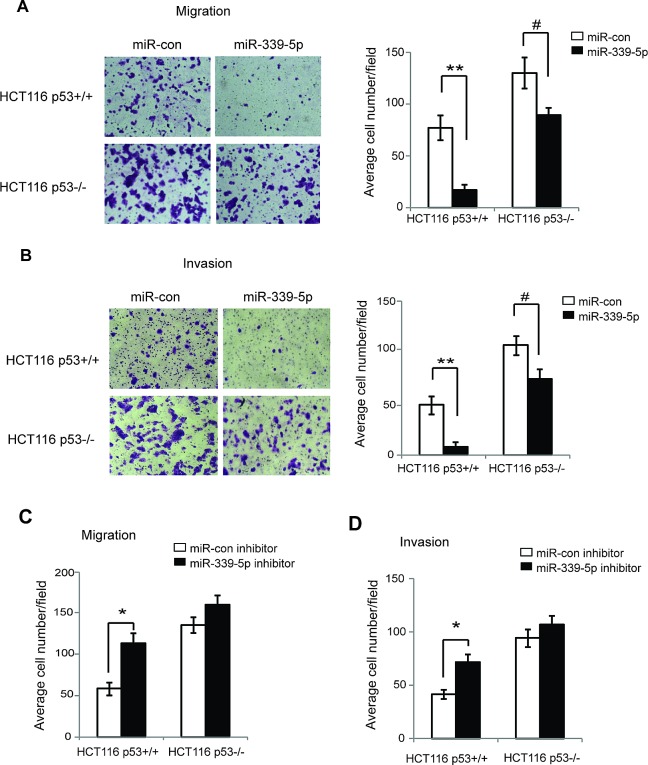
MiR-339-5p inhibits the migration and invasion of colorectal cancer cells in a largely p53-dependent manner (A) MiR-339-5p inhibited the migration of colorectal cancer cells in a largely p53-dependent manner. HCT116 p53+/+ and p53−/− cells transfected with miR-339-5p mimic or miR-con were seeded into chambers for migration assays. Left panels: Representative images of migrated cells; Right panel: Quantifications of average number of migrated cells per field. (B) MiR-339-5p inhibited the invasion of colorectal cancer cells in a largely p53-dependent manner. HCT116 p53+/+ and p53−/− cells transfected with miR-339-5p mimic or miR-con were seeded into matrigel-coated chambers for invasion assays. Left panels: Representative images of invading cells; Right panel: Quantifications of average number of invading cells per field. (C, D) The miR-339-5p inhibitor promoted the migration (C) and invasion (D) of colorectal cancer cells in a largely p53-dependent manner. HCT116 p53+/+ and p53−/− cells transfected with miR-339-5p inhibitor or miR-con inhibitor were used for migration and invasion assays as described in A and B, respectively. Data are presented as mean ± SD (n=3). #: *p*<0.05; *: *p*<0.01; **: *p*<0.001; two-tailed Student *t*-tests.

p53 plays an important role in inhibition of tumor metastasis [1, 4, 37]. Cell migration and invasion are two critical steps for tumor cell metastasis [38, 39]. Recently, miR-339-5p was reported to be associated with cancer metastasis and poor prognosis in cancer patients [25-27]. To investigate whether miR-339-5p inhibits tumor cell migration and invasion through the activation of p53, chamber transwell assays were employed to determine the effects of miR-339-5p on the abilities of cell migration and invasion in HCT116 p53+/+ and p53−/− cells. Compared with HCT116 p53+/+ cells, HCT116 p53−/− cells displayed enhanced abilities of migration and invasion (Figure 4A,B), demonstrating that p53 inhibited migration and invasion of tumor cells, which is consistent with previous reports [4, 37, 40, 41]. Notably, miR-339-5p greatly inhibited migration and invasion of HCT116 p53+/+ cells (Figure 4A,B). The inhibitory effects of miR-339-5p on migration and invasion were much less pronounced in HCT116 p53−/− cells (Figure 4A,B). Furthermore, the miR-339-5p inhibitor significantly promoted the migration and invasion of HCT116 p53+/+ cells, but displayed a much less pronounced effect on migration and invasion in HCT116 p53−/− cells (Figure 4C,D). Taken together, these results indicated that miR-339-5p inhibits migration and invasion of colorectal cancer cells *in vitro* through its activation of p53 function.

### MiR-339-5p inhibits colorectal tumorigenesis *in vivo* in a largely p53-dependent manner

**Figure 5:**
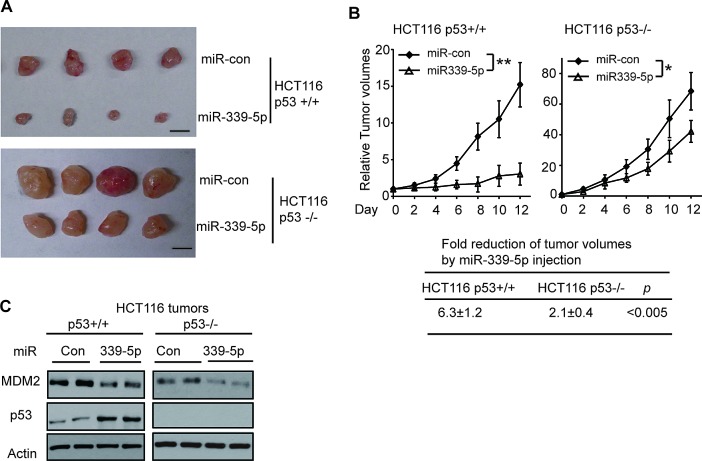
MiR-339-5p inhibits the growth of HCT116 xenograft tumors in a largely p53-dependent manner (A, B) MiR-339-5p inhibited the growth of HCT116 xenograft tumors in nude mice in a largely p53-dependent manner. Xenograft tumors were established by s.c. injection of HCT116 p53+/+ and HCT116 p53−/− cells into nude mice. When tumor volumes reached ~60mm^3^, tumors were injected with miR-339-5p mimic or miR-con once every two days for 6 times. (A) Representative tumors were photographed at 12 days after the first treatment with miR-339-5p mimic or miR-con. Scale bar: 10 mm. (B) Upper panels: The growth curves of HCT116 p53+/+ and p53−/− tumors after miR-339-5p injection. The relative volumes of the tumors before treatment at day 0 were designated as 1. Lower panel: The fold reduction of tumor volumes by miR-339-5p injection in both HCT116 p53+/+ and p53−/− tumors. Data are presented as mean ± SD (n=12 for each group). *: *p*<0.01; **: *p*<0.001; two-tailed Student *t*-tests. (C) Injection of miR-339-5p reduced MDM2 protein levels and increased p53 protein levels in HCT116 xenograft tumors. Six tumors from each group were used for western-blot analysis, and very similar results were observed. Represented are 2 tumors from each group.

p53 plays a critical role in tumor suppression, and loss of p53 promotes tumor growth[1, 2]. To investigate whether miR-339-5p inhibits the colorectal tumorigenesis *in vivo* through its activation of p53, HCT116 p53+/+ and p53−/− cells were injected (s.c.) into nude mice for xenograft tumorigenesis. When the tumor volume reached ~60 mm^3^, tumors were injected with miR-con or miR-339-5p mimic (once every 2 days for 6 times). As shown in Figure 5A&B, p53 loss promoted the growth of HCT116 tumors as demonstrated by the faster growth rate of HCT116 p53−/− tumors injected with miR-con compared with the HCT116 p53+/+ tumors injected with miR-con, which is consistent with previous reports [18, 42, 43]. Notably, compared with miR-con, miR-339-5p mimic greatly inhibited the growth of HCT116 p53+/+ tumors; the tumor volume was reduced by ~6 fold in HCT116 p53+/+ tumors (Figure 5A, B). This inhibitory effect of miR-339-5p on tumor growth was significantly less pronounced in HCT116 p53−/− tumors; the tumor volume was only reduced by ~2 fold in HCT116 p53−/− tumors (Figure 5A, B). Results from western-blot analysis confirmed that miR-339-5p mimic clearly reduced the MDM2 protein levels in both HCT116 p53+/+ and p53−/− tumors, and increased p53 protein levels in HCT116 p53+/+ tumors (Figure 5C). These results indicated that miR-339-5p inhibits tumor growth in a largely p53-dependent manner *in vivo* through its direct down-regulation of MDM2.

## DISCUSSION

MDM2 plays an important role in tumorgenesis [5, 9, 11]. As an E3 ubiquitin ligase, MDM2 interacts with p53 and mediates the degradation of the p53 protein. MDM2 overexpression and amplification is observed in many types of human cancers, including colorectal cancer, which contributes to tumorigenesis through attenuating p53 function [9-11]. In cells, many signals and factors can regulate the MDM2 protein, including genotoxic stress signals, oncogenic activation, ribosomal stress, chronic stress, neurohormones [11, 44, 45]. Recently, several miRNAs targeting MDM2 have been identified, including miR-192, miR-194, miR-25, miR-32 and miR-661, which revealed that miRNAs are a new component in the MDM2/p53 signaling pathway [20-22]. In this study, we found that miR-339-5p directly down-regulates MDM2 through binding to the 3′-UTR of *MDM2*. Through the negative regulation of MDM2, miR-339-5p increases p53 protein levels and functions, including p53 transcriptional activity, p53-mediated apoptosis and senescence in cells in response to stress. Thus, our results clearly demonstrated that miR-339-5p is an important negative regulator for MDM2 and a positive regulator for p53 in cells.

Recently, several studies reported that the expression of miR-339-5p was decreased in several different types of cancers, including colorectal cancer and breast cancer. Furthermore, lower levels of miR-339-5p were associated with cancer metastasis and poor prognosis in cancer patients [25-27]. These findings strongly suggest a potential role of miR-339-5p in tumor suppression. However, its mechanism is not well understood. In this study, we identified that MDM2 is a direct target for miR-339-5p. Through the negative regulation of MDM2, miR-339-5p increases p53 protein levels and functions. Considering the critical role of p53 in tumor suppression, our findings strongly suggest that activation of p53 is an important mechanism by which miR-339-5p displays a tumor suppressive function. Consistently, our results in this study showed that miR-339-5p inhibits the migration and invasion of colorectal cancer cells *in vitro* as well as the growth of colorectal xenograft tumors in mice in a largely p53-dependepnt manner through its repression of MDM2 expression. These results strongly suggest an important role of miR-339-5p in suppression of colorectal tumorigenesis through its regulation of the MDM2/p53 signaling.

Some interesting questions still remain unclear. For instance, the regulation of miR-339-5p in normal and tumor cells is largely unknown. It is unclear whether miR-339-5p itself can be regulated by stress signals in cells. It is also unclear why the expression of miR-339-5p is frequently down-regulated in different types of tumors. Our results showed that miR-339-5p inhibits tumor cell migration/invasion and xenograft tumor growth in a largely p53-dependenet manner. However, miR-339-5p also displays some p53-independent effects on both tumor cell migration/invasion and tumor growth, although these effects are much less pronounced compared with the p53-dependent effects of miR-339-5p. One miRNA can regulate many different targets [12-14]. Previous studies have identified several other miR-339-5p targets, including BCL-6 and PRL-1 [25, 26]. These identified targets and some additional unidentified targets for miR-339-5p could contribute to the p53-independent activities of miR-339-5p in tumor suppression. Furthermore, recent studies have reported that MDM2 also exhibits some p53-independent functions [9, 46]. The repression of MDM2 by miR-339-5p could also lead to the changes of activities and functions of other MDM2-regualted proteins in addition to p53, which may also contribute to the p53-independent activities of miR-339-5p in tumor suppression. Further studies are needed in the future to address these interesting questions.

In summary, our results in this study demonstrated that miR-339-5p negatively regulates MDM2 in colorectal cancer cells. Through the negative regulation of MDM2, miR-339-5p increases p53 protein levels and functions, including the p53 transcriptional activity and p53-mediated apoptosis and senescence in response to stress in colorectal cancer cells. Furthermore, miR-339-5p inhibits the migration and invasion of colorectal cancer cells *in vitro* and the growth of colorectal xenograft tumors *in vivo* in a largely p53-dependepnt manner. Thus, our results highlighted an important role of miR-339-5p in suppression of colorectal tumorigenesis through regulating the p53 activity and function.

## MATERIALS AND METHODS

### Cells and miRNA transfection

HCT116 p53+/+, HCT116 p53−/−, RKO p53+/+ and RKO p53−/− cells were generous gifts from Dr. Bert Vogelstein (John Hopkins University). H460 and MCF7 cells were obtained from ATCC. The miRNA mimic and miRNA inhibitor oligonucleotides (Ambion, TX) were transfected into cells using Oligofectamine (Invitrogen) as described [18].

### Construction of luciferase reporter vectors and luciferase reporter assays

The human *MDM2* 3′-UTR sequences (1580 bp, 38-1617 nt from the start of 3′-UTR) containing three putative miR-339-5p binding sites were amplified by PCR using following two primers: Forward primer 5′-ACT AGT TAT AAC CCT AGG AAT TTA GAC AAC C -3′ and Reverse primer 5′-AAG CTT ACA TCA TTA CTC CCA TCC CTT AC-3′. These two primers contain HindIII and SpeI recognization sites at the 5′ end of the primers, respectively. The PCR products were subcloned into the 3′ end of the pMIR-luciferase reporter vector (Ambion) at HindIII and SpeI sites. The mutations of three putative miR-339-5p binding sites were introduced using a Quikchange II XL Site-Directed Mutagenesis Kit (Stratagene/Agilent Technologies). Luciferase reporter assays were performed as previously described [18]. In brief, firefly pMIR-luciferase reporter vectors (100 ng) were transfected into cells in 6-well plates together with miR-339-5p mimic (100 nM) or scrambled miRNA mimic as a negative control by using Lipofectamine 2000. pRL-SV40 vectors (5 ng) that express *Renilla* luciferase (Promega) were co-transfected to normalize the transfection efficiency. Luciferase activities were measured at 24 h after transfection by using the Dual Luciferase Assay Kit (Promega).

### Western-blot assays

Standard western-blot assays were used to analyze protein expression as we previously described [47]. Following antibody were used: anti-MDM2 (2A10; generous gift from Dr. Arnold Levine) [48], anti-p53 (FL393, Santa Cruz Biotechonology), anti-p21 (Ab-1, EMD Millipore), anti-actin (#A5441, Sigma). The band intensity on western-blots was quantified by digitalization of the X-ray film and analyzed with ImageJ 1.45s software (NIH) and normalized to Actin.

### Taqman Real-Time PCR Analysis

Total RNA from cells was prepared by using an RNeasy kit (Qiagen). cDNA was prepared with random primers using TaqMan reverse transcription kit (Applied Biosystems) as we previously described [18]. Gene expression levels were determined by real-time PCR using Taqman PCR master mixture and primers. The expression of genes was normalized to actin gene.

### Cellular apoptosis and senescence analysis

Cellular apoptosis and senescence assays were performed as previously described [49]. For apoptosis analysis, cells were treated with 5-FU (300 μM), and collected at different time points (24 and 36 h) after treatment. Cells were washed with PBS, stained with a Alexa Fluor® 488 annexin V/Dead Cell Apoptosis Kit (Life Technologies) before being analyzed in a flow cytometer (Beckman Coulter). For senescence analysis, senescent cells were detected by using a Senescence β-Galactosidase Staining Kit (Cell Signaling) according to the manufacturer's protocol.

### Cell migration and invasion assays

The transwell systems (24 wells, 8 μM pore size, BD Biosciences) were used for cell migration and invasion assays as we previously described [50]. For migration assays, 5 ×10^4^ cells in 300 μl of serum-free medium were seeded into upper chambers, and the lower chamber was filled with 750 μl medium supplemented with 10% FBS. For invasion assays, transwell membranes were pre-coated with 50 μl Matrigel (BD Biosciences), and 1 ×10^5^ cells were seeded into upper chambers. After cells were cultured at 37 °C for 24 h (for migration assays) or 36 h (for invasion assays), cells in the upper surface of the membrane were removed and cells on the lower surface were fixed with methanol and stained with crystal violet. The number of cell was counted in at least five randomly selected fields under a microscope.

### Xenograft tumorigenicity assays

Xenograft tumorigenicity assays were performed as previously described with the approval of the Institutional Animal Care and Use Committee [47]. HCT116 p53+/+ and HCT116 p53−/− cells (5×10^6^ in 0.2 ml PBS) were injected (s.c.) into seven-week-old BALB/c nu/nu male athymic nude mice (Taconic, NY). When the volumes of xenograft tumors reached ~60 mm^3^, miR-339-5p mimic (0.5 nmol) or scrambled miRNA-con were injected directly into the tumors every two days for 6 times (n=12 per group), and mice were sacrificed at the day 12 (the day for the first injection was designated as day 0), and tumors were collected for analysis. Tumor volume = ½ (length × width^2^).

### Statistical analysis

The differences in tumor growth among groups were analyzed for statistical significance by ANOVA, followed by Student's *t*-test using a GraphPad Prism software. All other *P* values were obtained using two-tailed Student *t*-tests.
